# Impact of anti-inflammatories, beta-blockers and antibiotics on leaf litter breakdown in freshwaters

**DOI:** 10.1007/s11356-015-5798-3

**Published:** 2015-12-04

**Authors:** S. R. Hughes, P. Kay, L. E. Brown

**Affiliations:** School of Geography/water@leeds, University of Leeds, Leeds, West Yorkshire LS2 9JT UK; JBA Consulting Ltd, The Old School House, St Joseph’s Street, Tadcaster, North Yorkshire UK

**Keywords:** River, Water quality, Pollution, Pharmaceuticals, Emerging contaminants, Macroinvertebrates

## Abstract

Pharmaceuticals are now recognised as important pollutants in freshwater systems, but a shortcoming of effects studies is that they have focused on structural endpoints and impacts on ecosystem functioning are poorly understood. The decomposition of organic matter is an important functional process in aquatic systems, and it is known that this can be impacted by the presence of pollutants. Previous studies on leaf litter breakdown have only considered the effects of antibiotics and not other groups of drugs though. The current study investigated the effects of anti-inflammatories, a beta-blocker and an antibiotic on microbially mediated breakdown of leaf litter in the laboratory; colonisation of leaf packs by benthic macroinvertebrates when placed in a stream; and shredding of leaf litter by these organisms. Furthermore, the effects of single compounds relative to their mixture were assessed. It was found that exposure of leaf litter to the study compounds did not influence its breakdown by microbes in the laboratory or macroinvertebrates in a stream. Exposure of leaf litter to pharmaceuticals also had no effect on its colonisation by macroinvertebrates in this study. Many unknowns remain, however, and further studies of the effects of pharmaceuticals on structural and functional endpoints are needed to aid aquatic conservation.

## Introduction

The input of allochthonous carbon in the form of coarse particulate organic matter (CPOM; i.e. particles >1 mm in size) is a major and often dominant energy source in freshwater ecosystems (Webster et al. 1995; Giller and Malmqvist 1998). The decomposition of CPOM (including leaf litter) involves three processes: direct leaching of dissolved organic carbon (DOC), microbial decomposition and shredding by macroinvertebrates (Webster and Benfield 1986). These processes are interrelated, and mechanical shredding increases the surface area of leaf litter available for microbial colonisation (Giller and Malmqvist 1998). Colonisation by microbial communities can in turn affect mechanical breakdown due to influencing food selection by shredder macroinvertebrates. Leaf litter can be more palatable and have increased energy content for shredders as they consume both leaf material and the microbial colonisers (Giller and Malmqvist 1998; Graça et al. 2001). Studies have shown that fungi dominate over bacteria in these microbial communities and contribute most towards microbial decomposition rates (Gulis and Suberkropp 2003a, b). Without leaf litter decomposition, the associated carbon would be unavailable at higher trophic levels and downstream transport would be reduced (Giller and Malmqvist 1998). For example, disruptions to the input and processing of leaf litter during large experimental manipulations have been shown to decrease secondary production by up to 80 % (Wallace et al. 1997).

There are numerous studies documenting the impact of anthropogenic change on the decomposition rates of leaf litter in rivers (Feio et al. 2010; Medeiros et al. 2010). These studies demonstrate that leaf litter decomposition (including the colonisation by bacteria, fungi and macroinvertebrates) is sensitive to a wide range of environmental stressors. In particular, moderate nutrient enrichment appears to stimulate decomposition whereas high levels of nutrients, heavy metals or specific chemical compounds (e.g. pesticides) can be inhibitory. Studies have shown that leaf litter colonised under different pollutant loads, but then incubated under the same reference conditions, can decompose at different rates (Medeiros et al. 2010).

Pharmaceuticals are now recognised as significant pollutants of the aquatic environment worldwide, and their management is needed to conserve these habitats (Hughes et al. 2013). Studies of the effects of pharmaceuticals on freshwater ecosystem functioning are, nevertheless, sparse (Fent et al. 2006; Kümmerer 2009). and ecotoxicological research has, to a large extent, focused on standard acute tests measuring organismal-level metrics (e.g. feeding, growth, reproduction, mobility, mortality). Laboratory studies examining the effects of pharmaceuticals on leaf litter breakdown are rare (Hahn and Schulz 2007; Bundschuh et al. 2009). and none is available for compounds other than antibiotics. This study aimed to quantify the effects of exposure to five pharmaceutical compounds both in isolation and as a mixture on leaf litter decomposition during the microbial conditioning phase. Leaf packs were then moved to a stream and colonisation and breakdown by macroinvertebrates observed. Specific objectives were to

- Understand the effects of pharmaceutical compounds on the microbially mediated decomposition of leaf litter
- Examine the effects of leaf litter exposure to pharmaceuticals on colonisation by macroinvertebrates and in-stream decomposition
- Determine differences in effects when leaf litter is exposed to single pharmaceutical compounds and a mixture of these

## Methods

### Field site

**Fig. 1:**
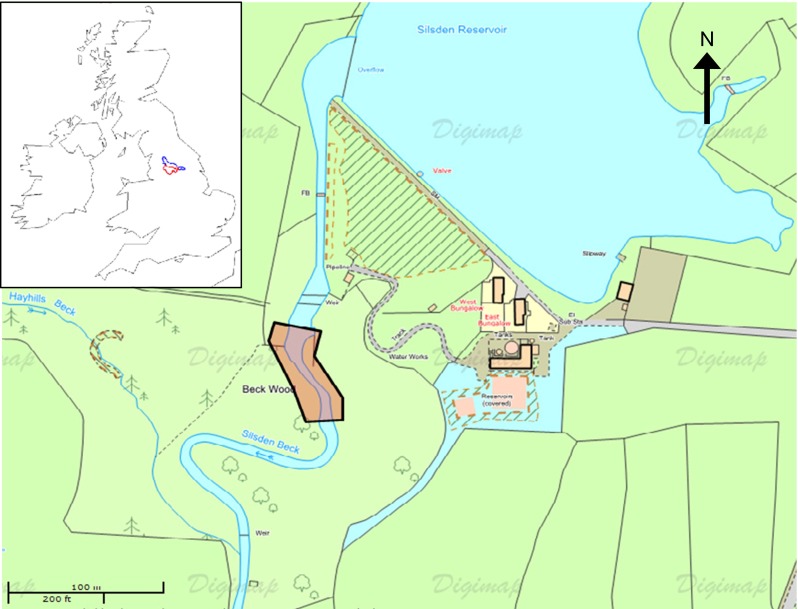
Site map of Silsden Beck, West Yorkshire, UK. The *highlighted area* indicates location of leaf pack placement. © Crown copyright/database. An Ordnance Survey/EDINA supplied service

The site selected for the in situ phase of the work was Silsden Beck, West Yorkshire (Fig. 1). The site is upstream of any wastewater treatment plant (WWTP) effluent inputs, thereby eliminating the potential for contamination by the study compounds which could have had direct impacts on the invertebrates present. A 1-L grab sample of water from the site was analysed by HPLC-MS/MS to confirm this and indicated that all five of the study compounds were below detection limits (5 ng L^−1^ for diclofenac, erythromycin, mefenamic acid and propranolol and 25 ng L^−1^ for ibuprofen). Wooded banks (predominantly beech, *Fagus sylvatica*) provided food to support a community of shredder macroinvertebrates (particularly *Gammarus pulex* (Crustacea: Amphipoda) and *Asellus aquaticus* (Crustacea: Isopoda). An initial survey found >50 individuals per kick sample; these were taken across the channel at five locations distributed evenly along a 100-m length of stream.

### Collection and preparation of leaf material

Abscised beech (*F. sylvatica*) leaves were collected adjacent to Silsden Beck and soaked in deionised water for 2 days to remove any extraneous soil or mineral matter on the surface of the leaves (which may affect mass loss calculations), prior to drying at 55 °C in an oven for 48 h (Benfield 2006). The leaves were then stored in sealed polythene bags in dark, dry conditions at room temperature until use. River water used in the laboratory experiments was collected from Silsden Beck into pre-cleaned 25-L plastic containers. This was filtered through a 0.064-mm sieve to remove any coarse sediment or invertebrates and stored at 7 °C in a controlled temperature room prior to use. This ensured that there was no difference in the extent of leaf conditioning across treatments at the start of the experiment as may have been the case if leaves were taken from the stream.

### Laboratory exposure of leaves to pharmaceuticals

Two-gram samples (±0.04 g) of dried beech leaves were placed within 1000-μm stiff mesh leaf packs (EFE UK Ltd., Cornwall, UK). Twelve artificial leaf packs were also prepared for in-stream placement in the same manner using 2-g samples of polyvinylchloride leaves that had been leached for 7 days in deionised water. This allowed consideration of whether the leaf packs were being colonised for their food value or merely the habitat that they provided. Twelve beech leaf packs were then randomly placed in each of 14 separate glass aquaria (45 × 38 × 30-cm Clear-seal aquaria; Clear-seal Ltd., Birmingham, UK) (total *n* = 168). The artificial leaf packs were split between the negative control and the high-concentration mixture. Each aquarium contained 20 L of water from Silsden Beck which was equilibrated for temperature and dissolved oxygen over 7 days prior to the addition of leaf packs. Throughout equilibration and exposure, the aquaria received constant aeration (Hailea ACO-9602 air pumps, Guangdong Hailea Group Co. Ltd., Guangdong, China) at 7 ± 0.3 °C and a light/dark period of 12:12 h.

After equilibration, treatment of 12 tanks with the pharmaceutical compounds was achieved by pipetting the appropriate volume of a working solution directly into the tank and then stirring thoroughly with a glass rod. A set of five study compounds (Table 1) was chosen based on risk quotients (RQ) (ratio of predicted or maximum environmental concentration to predicted no effect concentration) produced in previously published studies (Stuer-Lauridsen et al. 2000; Jones et al. 2002; Ferrari et al. 2004; Cleuvers 2005; Tauxe-Wuersch et al. 2005; Thompson 2006; Yamamoto et al. 2009). These studies used a combination of modelling and acute and chronic laboratory toxicity tests to assess impacts on fish, invertebrates and algae. A RQ ≥1 indicates the potential for impacts on aquatic organisms so this was used as the basis for selection. All pharmaceuticals were of the highest purity available (>95 %) and supplied by Sigma-Aldrich Company Ltd (Dorset, UK). Individual stock standard solutions were prepared on a weight basis in 100 mL of 100 % methanol and stored in the dark at −20 °C until used. A working mixture solution of all pharmaceuticals was prepared by appropriate dilution of the individual stocks in 100 % methanol. Nominal pharmaceutical concentrations were based on the maximum published concentrations in UK rivers (low treatment) (Ashton et al. 2004) which were multiplied by a factor of 1000 to yield the high treatment concentrations. These high concentrations would therefore be well in excess of any ever measured in the environment. Each tank was re-treated with working pharmaceutical solutions during the 8-week laboratory exposure period based on published half-lives in water (Tixier et al. 2003; Castiglioni et al. 2004; Löffler et al. 2005; Yamamoto et al. 2009). As the concentrations used were arbitrary and the aim of the experiment was to determine if effects could be observed rather than to establish specific dose-response relationships, they were not quantified by LC-MS. A solvent-only control (methanol) tank was also established in addition to a negative control.

**Table 1:** Study compounds and nominal exposure concentrations which leaf packs were exposed to in laboratory aquaria for 8 weeks before relocation to a stream

Compound	Concentration (ng L^−1^)	
	Low treatment	High treatment
Diclofenac (anti-inflammatory)	600	600,000
Erythromycin (antibiotic)	1000	1,000,000
Ibuprofen (anti-inflammatory)	5000	5,000,000
Mefenamic acid (anti-inflammatory)	400	400,000
Propranolol (beta-blocker)	200	200,000
Low-treatment mixture	Sum of above	n/a
High-treatment mixture	n/a	Sum of above

### In-stream placement of leaf packs

Following the laboratory exposure period, half of the leaf packs were relocated to Silsden Beck for 8 weeks and anchored randomly to the stream bed using house bricks held in place with steel bar. Suitable locations in the stream were identified as those deep enough to cover the whole leaf pack and with slower flows favoured by *Gammarus* and other shredders.

### Analysis of leaf decomposition

After retrieval from Silsden Beck, the leaf packs were opened and rinsed with deionised water onto a 0.064-mm sieve to remove any macroinvertebrates which were then preserved with 70 % methylated spirit for subsequent identification of taxa to family level under stereo microscope. The leaf packs were oven-dried at 55 °C for 48 h. A 0.5-g (±0.1 g) subsample of each leaf pack was combusted at 550 °C for 1 h in order to calculate ash-free dry mass (AFDM) and, therefore, % organic matter (%OM). This procedure was also followed for the leaf packs treated in the laboratory, but not moved to the stream, immediately after their removal from the treatment tanks. Handling loss control packs (*n* = 6) were also made and subjected to the same transport and handling procedures to account for potential losses during handling of the fragile dry leaf tissue (Benfield 2006). and the final mass changes for treatment packs were corrected as necessary.

### Data analysis

Data analysis was conducted in Minitab 16 (Minitab Inc., PA., USA) and PASW Statistics 17.0 (SPSS Inc., IBM). All data were first tested for normality using the Anderson-Darling test and homogeneity of variance using Levene’s test. The most powerful parametric (*t* test, ANOVA or general linear model) or non-parametric (Mann-Whitney or Kruskal-Wallis) statistical tests were then used. All proportional data were arcsine transformed prior to comparisons. Macroinvertebrate diversity indices (species richness, Bray-Curtis D) were calculated using Species Diversity and Richness v2.65 (Pisces Conservation, Lymington), and analysis of similarity (ANOSIM using Bray-Curtis distance over 9999 runs) was calculated using PAST (Hammer et al. 2001).

## Results

### Effect on leaf litter decomposition

**Fig. 2:**
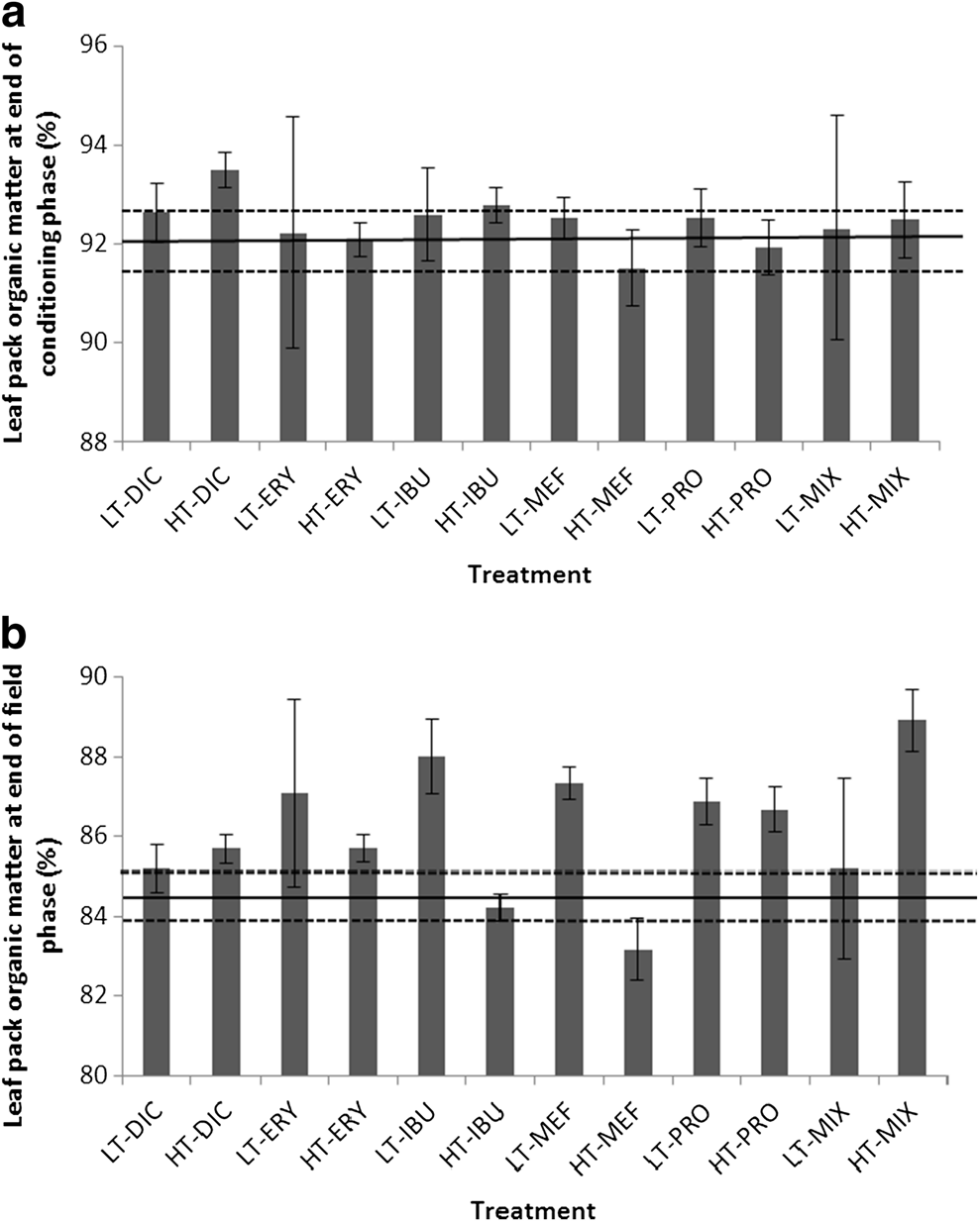
The organic matter content of leaf packs following an 8-week laboratory exposure to pharmaceuticals (**a**) followed by placement in a stream for a further 8 weeks (**b**). Initial leaf pack organic matter content was 94 %. *Error bars* represent one standard deviation. The *horizontal solid black lines* show the mean of the control leaf packs and the *dashed lines* one standard deviation. *LT* low treatment, *HT* high treatment, *DIC* diclofenac, *ERY* erythromycin, *IBU* ibuprofen, *MEF* mefenamic acid, *PRO* propranolol, *MIX* mixture

Exposure of leaf packs to any of the individual pharmaceuticals or their mixture in the laboratory did not result in a significant difference in leaf litter decomposition (*p* = 0.11) (Fig. 2). Similarly, no difference occurred whilst the leaf packs were subsequently located in a stream (*p* = 0.22).

### Impact on in-stream colonisation by macroinvertebrates

There was no significant difference in macroinvertebrate abundance or community composition across all treatments (*p* = 0.50) (Table 2). Mean total abundance in artificial leaf packs was lower than the *F. sylvatica* leaf packs although this difference was not significant (*p* = 0.14). The dominant taxon in all treatments was Chironomidae with smaller numbers of *A. aquaticus*, *G. pulex*, Oligochaeta, Plecoptera and Simuliidae. Of these, the abundance of *A. aquaticus* and *G. pulex* only is reported in Table 2, as these were the main shredders identified.

**Table 2:** Mean (±1 standard deviation) macroinvertebrate community metrics for leaf litter packs exposed to pharmaceuticals during an 8-week laboratory conditioning phase followed by placement for 8 weeks in a stream

Treatment	Total abundance	Taxonomic richness	Berger-Parker D (%)	Abundance *A. aquaticus*	Abundance *G. pulex*
ART	6.2 ± 4.6	2.8 ± 1.6	68.5 ± 19.8	0.5 ± 0.5	0.1 ± 0.3
CONT	12.2 ± 4.1	4.2 ± 0.8	63.7 ± 10.0	0.3 ± 0.5	0.2 ± 0.4
LT DIC	14.2 ± 7.6	3.2 ± 1.2	68.3 ± 16.9	0.5 ± 0.5	0.0 ± 0.0
HT DIC	11.5 ± 5.1	3.7 ± 1.4	57.2 ± 11.4	1.0 ± 1.7	0.0 ± 0.0
LT ERY	14.3 ± 9.3	3.2 ± 1.2	72.7 ± 16.4	0.7 ± 0.8	0.0 ± 0.0
HT ERY	14.0 ± 3.7	3.8 ± 0.4	64.6 ± 17.4	1.2 ± 1.3	0.4 ± 0.5
LT IBU	13.8 ± 5.3	4.0 ± 0.6	56.7 ± 11.1	1.0 ± 2.4	0.2 ± 0.4
HT IBU	17.5 ± 4.1	4.0 ± 1.8	58.1 ± 17.4	2.8 ± 3.1	0.3 ± 0.8
LT MEF	12.3 ± 9.0	4.2 ± 1.6	49.9 ± 13.5	1.5 ± 1.6	0.0 ± 0.0
HT MEF	13.3 ± 2.3	3.8 ± 1.3	70.7 ± 13.8	0.8 ± 0.8	0.0 ± 0.0
LT PRO	13.0 ± 6.3	3.7 ± 1.5	67.7 ± 19.6	0.3 ± 0.5	0.0 ± 0.0
HT PRO	13.7 ± 5.9	3.5 ± 0.5	64.2 ± 13.7	1.2 ± 1.2	0.2 ± 0.4
LT MIX	13.3 ± 6.3	4.8 ± 1.8	57.6 ± 9.8	1.5 ± 1.0	0.2 ± 0.4
HT MIX	12.5 ± 7.8	3.0 ± 1.7	79.6 ± 17.2	0.3 ± 0.5	0.3 ± 0.8

*ART* artificial leaf packs, *CONT* control, *LT* low treatment, *HT* high treatment, *DIC* diclofenac, *ERY* erythromycin, *IBU* ibuprofen, *MEF* mefenamic acid, *PRO* propranolol, *MIX* mixture

## Discussion

This was the first study to examine the effects of anti-inflammatories and beta-blockers on the important ecosystem functional process of leaf litter breakdown. It was also one of the first to study the impacts of antibiotics. No impacts were measured on leaf litter decomposition by microorganisms during laboratory conditioning with the study compounds or macroinvertebrates when the treated leaf packs were relocated to a stream. The currently available literature is inconclusive though. Hahn and Schulz (2007) found that the nutrient content of leaf packs was not affected by exposure to pharmaceuticals but that the leaf area consumed by *Gammarus* was. The authors therefore suggest that the reduced feeding rate may be due to the presence of the pharmaceuticals themselves rather than the extent of conditioning of the leaf material. As the leaf packs in the current study were only exposed to the study compounds in the laboratory phase of the experiment and not in the stream, this may explain why no difference in litter breakdown was observed. It is not, necessarily, easy to compare these two studies though due to the use of different study compounds, macroinvertebrates, exposure times and experimental set-up.

There is some ambiguity in the current study as to why the macroinvertebrates were colonising the leaf packs in the stream: for their food value, the habitat that they offered or both. Total abundance and taxonomic richness were lower in artificial leaf packs compared to those containing natural *F. sylvatica* leaves although the difference was not statistically significant. Moreover, data for other variables reported in Table 2 were similar which suggests that the habitat offered by the leaf packs may have been more important than their food value. The few other studies on the impacts of antibiotics on leaf litter breakdown have predominantly employed alder leaves (*Alnus glutinosa*), whereas we used *F. sylvatica* due to its dominance at the field site. It is unclear whether the leaf species used in the current study could have affected our results. Numerous studies on other pollutants have also used *F. sylvatica* (e.g. Dangles et al. 2004; Rasmussen et al. 2012a). These leaves have different nutritional characteristics which could result in differences in the extent to which they are colonised by biota (Graca 2001). Nevertheless, some work has found the chemistry of different leaf species to be unimportant in affecting their breakdown (Schindler and Gessner 2009; Frainer et al. 2015). It should also be considered that the use of only one mesh size may have affected the extent of colonisation by macroinvertebrates although any impact would have been the same across all treatments. The same size mesh has also been used in some previous studies by other workers (Jarvis et al. 2014).

The current study has suggested that a mixture of the pharmaceuticals studied does not represent any greater risk to leaf litter breakdown than the individual compounds, at least for leaf conditioning. The only other study to have investigated the impact of pharmaceutical mixtures on litter breakdown (Bundschuh et al. 2009) actually found an increase in their processing by *Gammarus fossarum*. This suggests that the pharmaceutical mixtures found in streams containing sewage effluent are not problematic for leaf litter conditioning and associated impacts on breakdown, as was found for single substances. This contrasts with studies of pharmaceutical compounds on structural endpoints which generally demonstrate effects following the pattern of concentration addition (CA) (Cleuvers 2003, 2004; Escher et al. 2005).

Other work has suggested that chemicals other than pharmaceuticals may exert a stronger influence in streams on litter breakdown. Englert et al. (2013) studied treated sewage effluent discharges, potentially containing a myriad of pollutants as well as pharmaceuticals, and found an impact on both leaf litter decomposition and colonisation by invertebrates. It was not determined, however, which chemicals were responsible for this effect. The results presented here suggest that compounds other than the five pharmaceuticals tested would have been the cause. Although exposure of aquatic mesocosms to carbamazepine resulted in significantly less organic matter in sediments, this was hypothesised to be due to changes in the shredding community rather than a direct impact on litter breakdown (Jarvis et al. 2014). Some studies have measured some significant impacts of pesticides on leaf litter consumption although a clear understanding does not exist (Rasmussen et al. 2012a, b). For instance, Flores et al. (2014) found that exposure of litter to an insecticide (diazinon) increased leaf litter breakdown whilst combined exposure with the fungicide, imazalil, resulted in a decrease. Other treatments (e.g. exposure to both compounds during the shredding phase only (by the amphipod, *Echinogammarus berilloni*) and not the conditioning phase) resulted in no deviation in litter breakdown from controls. This lack of clarity for a range of chemicals indicates that leaf litter breakdown is affected by a multitude of factors and that defining cause and effect relationships is very difficult.

## Conclusions

The current study suggests that concentrations of a range of pharmaceuticals found in river systems due to the release of sewage effluent do not represent a risk to leaf litter conditioning and its related breakdown by macroinvertebrates. Moreover, mixtures of the substances did not produce elevated effects. This is reassuring given the importance of this process for river ecosystem functioning. Other literature indicates that there may, however, be impacts due to direct effects of pharmaceuticals on invertebrates. There remain many unknowns as to the impact of pharmaceuticals on aquatic systems, and further studies of their effects on structural and functional endpoints are needed to address this knowledge gap and facilitate management of river ecosystems.
